# Occupation and oncology: cancer care in Palestine

**DOI:** 10.3389/fonc.2026.1895273

**Published:** 2026-09-04

**Authors:** Layth Mula-Hussain, Zahi Abdul-Sater, Caroline Samaha Jabbour, Fadi Atrash, Hibah Osman, Gevorg Tamamyan, Shada Wadi-Ramahi, Jemma Arakelyan, Khamis Elessi, Asem Mansour, Richard Sullivan

**Affiliations:** 1 Faculty of Medicine, The University of British Columbia, Vancouver, BC, Canada; 2 College of Public Health, Phoenicia University, Mazraat El Daoudiyeh, Lebanon; 3 Department of Radiation Oncology, Mount Lebanon Hospital, Beirut, Lebanon; 4 Radiation Oncology, Augusta Victoria Hospital, East Jerusalem, Palestine; 5 Supportive Oncology, Dana Farber Cancer Institute, Boston, MA, United States; 6 Institute of Cancer and Crisis, Yerevan, Armenia; 7 OncoDaily, Boston, MA, United States; 8 Yerevan State Medical University after Mkhitar Heratsi, Yerevan, Armenia; 9 Department of Radiation Oncology, University of Pittsburgh School of Medicine and UPMC Hillman Cancer Center, Pittsburgh, PA, United States; 10 Neurorehabilitation and Pain Medicine, Faculty of Medicine, University of Oslo, Oslo, Norway; 11 College of Medicine, Islamic University, Gaza, Palestine; 12 King Hussein Cancer Center, Amman, Jordan; 13 Institute of Cancer Policy, Kings College, London, United Kingdom

**Keywords:** cancer, oncology, occupation, Palestine, Israel, occupied Palestinian territory, West Bank, Gaza strip

## Abstract

Cancer care in fragile and conflict-affected settings is increasingly recognized as a challenge for global oncology. This Policy and Practice Review examines cancer care in Palestine as a case study of how prolonged occupation, armed conflict, restricted mobility, fragmented governance, fiscal constraint, and limited health sovereignty interact to shape early diagnosis, treatment continuity, radiotherapy access, systemic therapy, referral pathways, and patients’ rights. Rather than treating occupation as a uniform determinant of health outcomes, the manuscript advances a more defensible framework: cancer systems deteriorate where political control, poor governance, conflict, and fiscal crisis interrupt institutional continuity; conversely, health systems consolidate after conflict or occupation only when reconstruction is linked to restored civilian authority, accountable institutions, protected movement, and sustained infrastructure investment. Palestine is analyzed alongside brief historical and regional comparators, including Japan, West Germany, East Timor, Israel, and neighboring referral systems. The review highlights the absence of radiotherapy in the Gaza Strip, the late and still limited introduction of radiotherapy in the West Bank, dependence on permit-mediated referrals to East Jerusalem and regional centers, the collapse of oncology access after October 2023, and recent medicine shortages affecting cancer treatment in both the Gaza Strip and the West Bank. It connects oncology disruption with patients’ rights, structural violence, ecologies of war, therapeutic geographies, and health-system governance under occupation. Finally, it proposes practical actions: protected oncology referral corridors, urgent diagnostic recovery, chemotherapy and essential-medicine protection, companion-supported medical evacuation, radiotherapy reconstruction, audited cancer financing, regional mutual-aid agreements, digital oncology records, workforce support, and rights-based monitoring of delays, denials, and preventable deaths.

## Introduction

Cancer is a major global health burden, with nearly 20 million new cases and 9.7 million deaths estimated worldwide in 2022. Although improvements in prevention, early diagnosis, surgery, radiotherapy, systemic therapy, and palliative care have improved outcomes in many high-income settings, these gains remain unevenly distributed across regions and income groups (1, 2). The survival benefit of modern oncology depends on continuity: patients require timely pathology, staging imaging, surgery, chemotherapy, immunotherapy or targeted therapy when indicated, radiotherapy, supportive care, palliative care, survivorship monitoring, and longitudinal medical records. Interruptions at any point in this pathway can shift curable disease toward advanced or incurable disease.

Cancer care in fragile and conflict-affected settings is therefore especially vulnerable. The literature on cancer in conflict and humanitarian crisis shows that the problem is not only the destruction of hospitals, but also the erosion of referral governance, procurement systems, health financing, workforce retention, diagnostic quality, records, drug supply chains, and patients’ rights (3–6). Regional disparities are particularly important for Palestine, where access to comprehensive oncology services, advanced diagnostics, radiotherapy, immunotherapy, molecular testing, and specialized multidisciplinary care remains limited and heavily dependent on referrals (7). Before the most recent escalation, the Gaza Strip already faced a growing cancer burden and restricted access to diagnostic and treatment services (8).

This manuscript focuses on Palestine because its oncology crisis is shaped by a distinctive combination of prolonged occupation since 1967, territorial fragmentation, blockade, permit-mediated movement, dependence on external referrals, repeated military escalation, and internal Palestinian governance limitations. The Gaza Strip requires particular attention because repeated conflict, blockade, destruction of infrastructure, and loss of health workers compound an already fragile cancer system. An emerging public-health perspective has also hypothesized that environmental contamination from military action, destruction-related waste, and prolonged dependence on nutritionally inadequate food sources may contribute to future increases in cancer incidence, while simultaneous diagnostic delay may increase the proportion of advanced and less treatable cancers (9).

This article is structured as a policy-oriented narrative review. It uses Palestine as the detailed case study and employs brief historical and regional comparators as analytic contrasts rather than as direct analogues. The aim is to clarify how occupation, armed conflict, restricted movement, health-sovereignty constraints, governance integrity, and institutional continuity interact to shape cancer care. The manuscript also seeks to move from description toward practical strategies that can be used by clinicians, humanitarian agencies, regional cancer centers, donors, and policymakers.

## Materials and methods

This manuscript is a narrative policy and practice review integrating historical, oncology, governance, humanitarian-health, and global-health literature related to cancer care in Palestine. Data sources included peer-reviewed publications, reports from the Palestinian Ministry of Health, the World Health Organization (WHO), the International Agency for Research on Cancer (IARC), the International Atomic Energy Agency (IAEA), United Nations agencies, Transparency International, the World Bank Worldwide Governance Indicators (WGI), and Global Burden of Disease health-system indicators (10–14). Comparative oncology-system indicators were synthesized across selected settings, including Palestine, Israel, Jordan, Egypt, Lebanon, Japan, Germany, and East Timor.

For terminology, this manuscript uses the term “occupied Palestinian territory” (oPt) when referring to the West Bank, including East Jerusalem, and the Gaza Strip in the legal and United Nations sense. The term “Palestine” is used when referring to the population, health system, and national cancer-control context. The term “the Gaza Strip” is used throughout to avoid conflating the geographic territory with Gaza City.

Mortality-to-incidence ratios (MIRs) were calculated by the authors using age-standardized mortality and incidence rates from GLOBOCAN 2022 according to the formula: MIR = age-standardized mortality rate/age-standardized incidence rate (12). MIR is used here only as an ecological proxy for cancer-system performance. Its interpretation in Palestine is limited by incomplete cancer registration, under-ascertainment of incidence, variable capture of deaths, disruption of reporting systems, and especially weak data completeness for the Gaza Strip. Accordingly, the Palestinian MIR should not be interpreted as a precise country-to-country estimate; it is better understood as an approximate and likely conservative signal of outcome disparity in a setting where true incidence, stage distribution, treatment abandonment, and mortality may be incompletely captured.

Radiotherapy machine-density estimates were derived from IAEA DIRAC and supplemented by facility-level information reported by Palestinian oncology providers and publicly available institutional sources (32–34). Because machines in East Jerusalem and the West Bank are not equally accessible to all Palestinians, especially patients from the Gaza Strip, the machine-density figures should be interpreted as approximate installed or potentially available capacity rather than effective patient access.

Corruption Perceptions Index (CPI) scores were taken from Transparency International and used only as broad contextual indicators of public-sector governance; they do not measure occupation, fiscal coercion, clinical quality, or health-system accountability directly (11). WGI government-effectiveness and health-care access indicators were similarly treated as contextual variables, not causal proof (13, 14). The manuscript does not aim to establish direct causal inference between occupation, governance, and oncology outcomes. Instead, it examines how external control, internal governance capacity, fiscal constraints, armed conflict, and institutional continuity interact to affect cancer-care delivery.

Comparator selection was purposeful. Japan and West Germany were selected not because they are close analogues to Palestine, but because they demonstrate that occupation followed by sovereignty restoration, reconstruction, and institution-building may be followed by health-system consolidation. East Timor was added as a lower-income post-conflict comparator because it illustrates how health-system recovery after prolonged conflict depends on domestic stewardship, donor coordination, and transition from emergency relief to government-led services. Other nations, like Kosovo and Western Sahara, were considered but not used as central comparators because the available oncology-system data are less directly comparable to the Palestinian referral, radiotherapy, and registry questions addressed here. These comparisons are therefore heuristic, not causal.

## Conceptual framework: occupation is not destiny, but institutions matter

Occupation and external control can produce divergent health-system trajectories. In some settings, occupation or colonial governance has been associated with exploitation, segregation, and underdevelopment of local health infrastructure; in others, occupying or mandate powers introduced sanitation, epidemiological surveillance, hospitals, or public-health administration, often in ways that also served military or administrative objectives (15, 16). Post-war Japan and West Germany represent a distinct pattern: military occupation was time-limited, embedded in large reconstruction architecture, and followed by restored sovereignty, institution-building, fiscal investment, and integration into Cold War political economies (17, 18).

The structural differences from Palestine are substantial and must be made explicit. Japan and West Germany were defeated industrial states with pre-existing bureaucratic capacity, extensive external reconstruction investment, strategic geopolitical value to the United States and its allies, and a relatively clear transition from military occupation to internationally recognized sovereignty. Palestine, by contrast, remains territorially fragmented, lacks sovereign control over borders, movement, airspace, imports, currency, and much of its fiscal base, and has not undergone a comparable reconstruction settlement. East Timor offers a more relevant lower-income contrast: after decades of conflict and Indonesian occupation, health-system rehabilitation between 1999 and 2002 depended on the creation of an Interim Health Authority, transition from NGO-led emergency relief to local stewardship, and coordinated donor support (19). These examples therefore do not show that occupation is benign; they show that oncology systems require political transition, protected institutions, and accountable reconstruction.

Palestine differs in a crucial respect. The Israeli occupation of the West Bank, including East Jerusalem, and the Gaza Strip began in June 1967 according to United Nations usage (20, 21). Unlike time-limited post-war occupations followed by restored sovereignty, Palestinian health governance has remained territorially fragmented and externally constrained for more than five decades. Some early public-health indicators improved during the first decades after 1967 through vaccination, labor-market access, and partial integration with Israeli systems (22, 23). However, these gains occurred within a framework lacking sovereign control over borders, movement, infrastructure planning, and procurement; post-Oslo health-sector development also remained constrained by structural barriers to institutional development (24).

Oncology exposes the weakness of dependency because cancer care cannot rely on intermittent humanitarian exceptions. It requires diagnostic capacity, repeated treatment visits, reliable drug supply, radiation-safety infrastructure, functioning referral payment systems, multidisciplinary planning, and protected patient movement. Palestine is therefore not an exception to a general occupation rule; it is a case in which prolonged occupation, blockade, fiscal dependency, internal political division, and repeated armed conflict converge to make cancer care structurally fragile (**Table 1**).

**Table 1:** Analytical framework: determinants of cancer-care resilience in occupied and conflict-affected settings.

Determinant	Mechanism affecting oncology	Palestinian example	Policy implication
Occupation and mobility control	Permit systems, checkpoints, border closures, and separation of treatment sites interrupt timed care.	Patients from the Gaza Strip require referral for radiotherapy and complex cancer care.	Protected referral corridors, time-bound decisions, and appeal mechanisms.
Health sovereignty	Control over imports, infrastructure, financing, workforce, and planning determines local capacity.	Radiotherapy, medicines, equipment procurement, and referral payments remain externally constrained.	Recognize oncology infrastructure and drug procurement as sovereignty-linked health capacity.
Governance and fiscal integrity	Weak accountability, factional division, and fiscal crisis reduce efficient use of health spending and donor investment.	Referral debts, delayed reimbursements, and medicine shortages affect care in the West Bank and Gaza Strip.	Audited oncology budgets, transparent procurement, and public reporting of stock-outs and referrals.
Armed conflict	Destruction of hospitals, laboratories, power systems, transport, and workforce continuity interrupts diagnosis and treatment.	Post-2023 disruption of hospitals, pathology, chemotherapy, and referrals in the Gaza Strip.	IHL (international humanitarian law) protection, diagnostic recovery, and reconstruction planning.
Regional cooperation	Neighboring systems absorb patients during crises but can be overwhelmed or inaccessible.	East Jerusalem, the West Bank, Jordan, Egypt, Israel, and Lebanon are referral nodes.	Pre-negotiated mutual-aid agreements with financing, data-sharing, and companion support.

## Palestine: divergent health trajectories after 1948 and 1967

Before 1948, healthcare services in Mandatory Palestine were uneven and limited, delivered through a combination of British administration, missionary hospitals, charitable institutions, and emerging community institutions (25). After 1948, the establishment of Israel and the displacement of approximately 700,000 Palestinians during the Nakba disrupted Palestinian social, economic, and medical infrastructure (26). At the same time, Israel’s health system developed through immigration, pre-state institutional capacity, sick funds, university hospitals, philanthropy, later state insurance, and biomedical research systems (27, 28).

From 1948 to 1967, the Gaza Strip was under Egyptian administration and the West Bank, including East Jerusalem, was under Jordanian control. Palestinian health services remained fragmented, while Israel progressively developed advanced tertiary and oncology infrastructure (27, 28). The June 1967 war created a new health-system reality in which access, movement, imports, and referral routes for Palestinians were increasingly shaped by military administration and later by permit regimes. Movement restrictions became important after 1967, intensified during the First and Second Intifadas, and became especially restrictive for the Gaza Strip after the blockade imposed from 2007 onward (29–31). The geographic and legal context is now referenced using United Nations cartographic material rather than non-authoritative maps (20).

Radiotherapy is one of the clearest markers of inequity. The Gaza Strip has no functioning radiotherapy service. For years, Palestinian patients requiring radiotherapy depended on referral to East Jerusalem, Israel, Egypt, Jordan, or other centers. East Jerusalem’s Augusta Victoria Hospital has long served as the main Palestinian radiotherapy hub, but reaching it from the Gaza Strip or parts of the West Bank is a political and administrative process, not only a medical referral (29, 30, 33).

The West Bank has not had long-standing radiotherapy autonomy. Until very recently, local radiotherapy options were essentially absent outside East Jerusalem. Two additional West Bank services have recently begun functioning, including a private center in Ramallah and a center affiliated with An-Najah National University Hospital, each with limited machines, specialist staffing, and referral capacity (34). This late introduction of radiotherapy in the West Bank highlights the structural inequity compared with Israel, where radiotherapy and advanced oncology services have been established for decades. Moreover, installed machines do not automatically translate into access: government referrals, insurance eligibility, hospital reimbursement delays, workforce shortages, checkpoint delays, and the near-total inability of Gaza Strip patients to reach the West Bank after October 2023 substantially limit effective radiotherapy access (32–35).

## Governance under occupation: accountability, fiscal control, and cancer outcomes

The relationship between governance and health outcomes is well documented. Cross-country analyses show that corruption and weak governance are associated with lower health-spending efficiency and poorer population health outcomes (36). Hanf and colleagues estimated that corruption may be indirectly associated with substantial avoidable child mortality worldwide, while Rajkumar and Swaroop showed that public health spending has stronger effects where governance quality is better (37, 38). In oncology, Mostert and colleagues argued that corruption can distort procurement, access, donor returns, and cancer-care delivery (39).

The Palestinian case should not be framed as a competition between occupation and governance. Occupation weakens governance by limiting fiscal autonomy, movement, procurement, infrastructure planning, and the ability to unify services across the West Bank, East Jerusalem, and the Gaza Strip. At the same time, internal Palestinian governance weaknesses, factional division, opaque referral spending, and limited accountability can further reduce the effectiveness of scarce oncology resources (40). A defensible analysis must therefore treat governance and occupation as interacting determinants rather than separate explanations.

This interaction has become clinically visible in the medicine-supply crisis. OCHA reported in June 2026 that the Palestinian Ministry of Health warned of rapidly worsening shortages of medicines, laboratory supplies, and medical consumables, which it attributed to prolonged withholding of Palestinian clearance revenues. More than one-third of essential medicines had reached zero-stock levels, including 180 of 520 essential medicines and 50 of 97 cancer treatment drugs; the Ministry warned that more than 4,000 cancer patients and thousands of dialysis patients were at risk, and that more than 11,000 scheduled surgeries had been postponed since the beginning of 2026 (41). These figures illustrate how externally imposed fiscal constraint and internal system fragility converge: the collapsed system in the Gaza Strip cannot be compensated for by a West Bank health system that is itself financially and materially strained.

CPI data provide contextual but incomplete governance information. In the 2025 CPI, Germany scored 77, Japan 71, Israel 62, Jordan 50, Egypt 30, and Lebanon 23; Palestine was not rated because it did not meet the minimum data-source requirements (11). These scores should not be used to compare the morality or political legitimacy of states, nor to imply that corruption alone explains Palestinian cancer outcomes. Rather, they reinforce a narrower point: cancer systems depend on stable institutions, transparent procurement, and reliable financing. In Palestine, those institutional requirements are weakened simultaneously by occupation-linked constraints and internal governance limitations (**Table 2**).

**Table 2:** Comparative indicators relevant to cancer-system resilience. Indicators are contextual and should not be interpreted as causal proof.

Setting	Why included	Selected governance/health-system indicators	Radiotherapy/access context	Interpretation
Japan	High-income post-war occupation followed by sovereignty restoration and reconstruction.	CPI 2025: 71; HAQ and WGI high.	Approx. 7.8 RT machines/million.	Demonstrates health-system consolidation after occupation when reconstruction and sovereignty are restored; not a direct analogue.
West Germany	High-income post-war occupation followed by federal institution-building and sovereignty restoration.	CPI 2025: 77; HAQ and WGI high.	Approx. 7.0 RT machines/million.	Shows that time-limited occupation plus reconstruction differs structurally from prolonged occupation without sovereignty.
East Timor	Lower-income post-conflict transition after prolonged occupation and violence.	Post-conflict recovery depended on local stewardship and donor coordination.	Limited advanced oncology infrastructure during early recovery.	More relevant to fragile-state reconstruction; highlights need for domestic governance and coordinated transition.
Israel	Neighboring occupying power and regional oncology reference point.	CPI 2025: 62; HAQ and WGI high relative to regional comparators.	More than 5 RT machines/million; advanced oncology infrastructure.	Demonstrates regional capacity contrast and inequitable access across political boundaries.
Jordan, Egypt, Lebanon	Regional referral and overflow systems for Palestinian patients.	CPI 2025: Jordan 50, Egypt 30, Lebanon 23.	Variable RT and systemic-therapy capacity; Lebanon is conflict-fragile and refugee-hosting.	Regional mutual aid is essential but cannot substitute for local Palestinian capacity.
Palestine/oPt	Detailed case study of prolonged occupation, blockade, fiscal constraint, and fragmented governance.	Palestine not rated by CPI; WGI and HAQ affected by incomplete sovereignty and data limitations.	The Gaza Strip has no RT; West Bank RT began recently and remains limited; effective access depends on movement and referral financing.	Cancer-system fragility reflects interacting external control, internal governance limits, and repeated conflict.

## The Gaza strip: from restricted access to system collapse

Before October 2023, patients from the Gaza Strip who required radiotherapy, complex surgery, hematopoietic-cell transplantation, advanced imaging, or some systemic therapies often depended on travel to East Jerusalem, the West Bank, Israel, Egypt, or Jordan. WHO access reports documented delays and denials in permit applications, and rights-based analyses described how permit delays, denials, and companion denials disrupted care (30, 35, 42, 43). The evidence base is heterogeneous and consists mainly of access monitoring, rights-based documentation, and observational reports rather than randomized or prospective epidemiological studies. The manuscript therefore describes these sources as evidence of reported associations and clinically plausible harm, not as definitive causal estimates of mortality.

After October 2023, the referral system itself was severely disrupted. Hospitals faced damage, evacuation orders, fuel shortages, electricity failure, stock-outs, loss of diagnostic and pathology capacity, and extreme constraints on patient evacuation. MSF and ICRC documented the broader collapse of health services, while oncology-specific consequences include missed radiotherapy, interrupted chemotherapy, loss of staging imaging, pathology disruption, limited blood products, absence of analgesics, delayed diagnosis of new cases, and the loss or fragmentation of records (44, 45).

The Gaza Strip illustrates the difference between a health system under strain and a health system whose oncology functions become nearly impossible to deliver. The concept of therapeutic geographies helps define this reality: cancer care is geographically reorganized by checkpoints, borders, military operations, permits, refugee status, and geopolitics. Patients may need to navigate the Gaza Strip, Erez/Beit Hanoun, East Jerusalem, the West Bank, Jordan, Egypt, Israel, or Lebanon, depending on treatment type, funding, permit status, and security context. In oncology, where delayed surgery, missed radiation fractions, and interrupted chemotherapy cycles can alter prognosis, geography becomes biology (46).

## Structural violence, ecologies of war, and patients’ rights

Structural violence describes how social and political systems produce injury without direct physical assault (47). In cancer care, structural violence appears as delayed biopsy, interrupted chemotherapy, lack of radiotherapy, loss of medical records, drug shortages, inaccessible opioids, and collapsed referral pathways. Its effects extend beyond battlefield injury to food insecurity, scarcity of clean water, destroyed health infrastructure, and malnutrition that can shape long-term population health (50).

Ghassan Abu-Sittah’s framing of the ecologies of war adds that war reorganizes the entire environment necessary for survival: hospitals, electricity, water, fuel, roads, supply chains, and the social conditions that make healing possible (48). Rita Giacaman’s work on health, chronic war-like conditions, and human security in the occupied Palestinian territory similarly shows that health is inseparable from political determinants, dignity, mobility, and the capacity to live ordinary life under prolonged constraint (22, 49).

Cancer care is also a patients’ rights and human rights issue. Patients have rights to timely diagnosis, informed participation in treatment decisions, privacy, pain relief, palliative care, continuity of care, and equitable access to multidisciplinary services (51, 52). The right to the highest attainable standard of health is undermined when a patient cannot access radiotherapy because a border is closed, cannot continue chemotherapy because medicines are unavailable, cannot obtain staging because imaging equipment is damaged, or dies while awaiting a permit or evacuation. Framing oncology as a rights issue does not politicize cancer care; it restores the ethical and legal language needed to protect patients whose disease progression does not pause for war.

## Health sovereignty and the counterfactual of non-occupation

Theoretically, in the absence of occupation and blockade, cancer care in Palestine could evolve from a fragmented, permit-dependent referral system into a national cancer-control program integrating the Gaza Strip, the West Bank, and East Jerusalem. Such a model would allow coordinated development of diagnostic facilities, pathology networks, radiotherapy infrastructure, cancer registration, screening programs, protected oncology drug procurement, palliative-care services, and longitudinal follow-up systems.

This counterfactual should not be romanticized. Sovereignty alone would not eliminate financing constraints, workforce shortages, political instability, corruption risks, or regional economic pressures. However, it would remove one of the most consequential determinants of Palestinian cancer outcomes: dependence of diagnosis and treatment continuity on movement permits, border closures, military escalation, and externally controlled referral pathways. Restored health sovereignty would therefore be necessary but not sufficient; governance integrity, factional reconciliation, transparent oncology budgeting, and accountable institution-building would remain prerequisites for converting sovereignty into durable cancer-system performance.

## Practical action plan

A policy-relevant review must move beyond diagnosis toward operational recommendations. The first priority is not a single modality, but restoration of the cancer pathway from diagnosis to treatment completion. For the Gaza Strip, the most urgent early reconstruction need is diagnostic recovery: functioning laboratories, pathology, imaging, blood services, basic surgical capacity, medical records, and safe referral triage. Without diagnostic capacity, radiotherapy and systemic therapy cannot be delivered safely or appropriately.

Second, protected oncology referral corridors should be negotiated under international humanitarian law for patients requiring urgent treatment abroad, including radiotherapy, pediatric oncology, complex surgery, hematopoietic-cell transplantation, advanced imaging, and essential systemic therapy. These corridors should include time-bound decisions, transparent appeals, neutral monitoring, financial support at the receiving destination, and permission for a medically and socially appropriate companion when needed. Securing travel is not a novel solution, but it must be implemented as a complete care pathway rather than a one-way evacuation event.

Third, all cancer-treatment modalities must be protected. While radiotherapy availability is a visible marker of structural inequity, chemotherapy, antiemetics, analgesics, blood products, antibiotics, laboratory reagents, and palliative-care medicines are equally essential. The OCHA-reported shortage of cancer drugs in the West Bank and the near-collapse of oncology supply in the Gaza Strip demonstrate that drug availability is now a central cancer-control emergency (41).

Fourth, radiotherapy reconstruction should be treated as core health infrastructure rather than a luxury. The Gaza Strip requires phased radiotherapy capacity planning only after minimum diagnostic, safety, electricity, engineering, and workforce conditions are in place. Radiotherapy development requires protected bunker design, redundant power supply, service contracts, spare-part logistics, radiation-safety regulation, dosimetry, quality assurance, and workforce training for radiation oncologists, medical physicists, dosimetrists, radiation therapists, oncology nurses, and engineers. In settings where electricity and maintenance are unstable, technology selection must match resilience needs and safety standards, using WHO/IAEA procurement guidance rather than symbolic equipment donation (53).

Fifth, cancer financing should be audited. Donor support and government referral spending should be linked to transparent oncology budgets, drug availability, referral payment timelines, registry completeness, procurement safeguards, and measurable health-sector outcomes.

Sixth, regional mutual-aid agreements should formalize referral roles among East Jerusalem, the West Bank, Jordan, Egypt, Israel, Lebanon, and other regional centers, with predefined costs, transport routes, data-sharing rules, and crisis activation mechanisms. Such mechanisms should also account for Palestinian refugees in neighboring countries, including Lebanon, whose access to care is shaped by socioeconomic vulnerability (54).

Seventh, digital oncology records, cloud-based tumor boards, pathology teleconsultation, and cross-border case navigation should reduce loss of medical history during displacement and referral. Eighth, diaspora oncologist, physicist, nurse, pharmacist, and navigator rotations should be organized through structured platforms such as OncoCorridor, linked to local capacity-building rather than episodic missions (55). Recent radiation oncology literature similarly frames the survival of radiotherapy services in conflict-affected regions as a global responsibility requiring education, workforce development, technology support, research, advocacy, and long-term resilience planning (56).

Finally, the framework should remain explicitly rights-based and clinically grounded. The clinical policy goal is to reduce preventable cancer deaths by protecting diagnosis, movement, treatment continuity, essential medicines, radiotherapy access, palliative care, and data systems. The political context cannot be ignored, but in a medical journal it is most defensibly expressed through its direct consequences for patient access, health sovereignty, and the right to timely and dignified cancer care (**Table 3**).

**Table 3:** Practical action plan for cancer care in Palestine.

Priority	Action	Expected benefit
Diagnostic recovery	Rebuild laboratories, pathology, imaging, blood services, medical records, surgical triage, and referral navigation in the Gaza Strip.	Earlier diagnosis, safer treatment selection, fewer empiric or delayed decisions.
Protected referrals and evacuation	Create time-bound monitored corridors for radiotherapy, complex surgery, pediatric oncology, systemic therapy, imaging, and companions.	Reduced delays, denials, treatment abandonment, and preventable mortality.
Chemotherapy and essential medicines	Protect supplies of chemotherapy, immunotherapy when indicated, analgesics, antiemetics, antibiotics, blood products, and palliative medicines.	Continuity of systemic therapy and symptom control.
Radiotherapy reconstruction	Plan resilient RT capacity with trained staff, power redundancy, service contracts, spare parts, radiation safety, and quality assurance.	Reduced long-term dependence on permits and external referrals.
Audited financing	Tie donor and public support to transparent oncology budgets, procurement, registry completeness, and referral payment timelines.	Better conversion of scarce funds into treatment access.
Regional mutual aid	Formalize agreements with East Jerusalem, West Bank centers, Jordan, Egypt, Israel, Lebanon, and other regional centers.	Predictable care during crises and overflow.
Digital oncology	Use cloud-based records, tele-oncology, pathology networks, and cross-border tumor boards.	Reduced loss of records and faster treatment decisions during displacement.
Rights monitoring	Document denied permits, interrupted therapy, medicine stock-outs, preventable deaths, pain-control failures, and breaches of dignity or consent.	Accountability and patient-centered reform.
Health sovereignty and freedom of movement	Frame movement, infrastructure protection, procurement, and financing as clinical requirements for cancer care continuity.	Moves policy from crisis management toward durable cancer-system resilience.

## Conclusion

This policy review shows how occupation, armed conflict, governance integrity, fiscal control, and institutional continuity interact to determine cancer-care outcomes. The revised framing avoids treating occupation as destiny or governance as a competing explanation. Instead, it shows that cancer systems are most vulnerable when external control, movement restrictions, fiscal dependency, internal fragmentation, and repeated conflict disrupt the institutions required for diagnosis and treatment continuity.

Cancer care in Palestine has been made fragile by the interaction of restricted movement, limited health sovereignty, internal governance challenges, underdeveloped radiotherapy and diagnostic capacity, essential-medicine shortages, and repeated armed conflict. The Gaza Strip’s lack of radiotherapy is not merely an equipment gap; it is one expression of a system in which borders, electricity, referral payments, procurement, workforce development, diagnostics, and patients’ rights are inseparable.

A patient-centered cancer strategy should therefore combine immediate humanitarian protection with long-term institution-building: diagnostic recovery, protected referrals, chemotherapy and essential-medicine supply, palliative-care access, radiotherapy and registry capacity, audited financing, and accountable governance. While political status questions cannot be adjudicated by an oncology review, the authors’ position is that durable improvement in Palestinian cancer outcomes requires freedom of movement, protection of health infrastructure, and health sovereignty because these are practical requirements for timely, continuous, and dignified cancer care.
